# The role and mechanisms of macrophage polarization and hepatocyte pyroptosis in acute liver failure

**DOI:** 10.3389/fimmu.2023.1279264

**Published:** 2023-10-26

**Authors:** Dan Xie, Shi Ouyang

**Affiliations:** Key Laboratory of Biological Targeting Diagnosis, Therapy and Rehabilitation of Guangdong Higher Education Institutes, Department of Infectious Diseases, The Fifth Affiliated Hospital of Guangzhou Medical University, Guangzhou, China

**Keywords:** acute liver failure (ALF), macrophage, polarization, pyroptosis, immune

## Abstract

Acute liver failure (ALF) is a severe liver disease caused by disruptions in the body’s immune microenvironment. In the early stages of ALF, Kupffer cells (KCs) become depleted and recruit monocytes derived from the bone marrow or abdomen to replace the depleted macrophages entering the liver. These monocytes differentiate into mature macrophages, which are activated in the immune microenvironment of the liver and polarized to perform various functions. Macrophage polarization can occur in two directions: pro-inflammatory M1 macrophages and anti-inflammatory M2 macrophages. Controlling the ratio and direction of M1 and M2 in ALF can help reduce liver injury. However, the liver damage caused by pyroptosis should not be underestimated, as it is a caspase-dependent form of cell death. Inhibiting pyroptosis has been shown to effectively reduce liver damage induced by ALF. Furthermore, macrophage polarization and pyroptosis share common binding sites, signaling pathways, and outcomes. In the review, we describe the role of macrophage polarization and pyroptosis in the pathogenesis of ALF. Additionally, we preliminarily explore the relationship between macrophage polarization and pyroptosis, as well as their effects on ALF.

## 1 Introduction

The liver plays a crucial role in the immune system by serving as a vital organ responsible for removing toxins, producing immune proteins, and maintaining metabolic homeostasis (1). The first point to note is that the liver contains a high concentration of both innate and adaptive immune cells. These cells have the ability to trigger inflammation and liver damage in response to disease, but also have the capability of maintaining a state of tolerance during homeostasis. The liver is home to a variety of T cell subsets, including regulatory T cells and cytotoxic T lymphocytes, which play an essential role in maintaining liver tolerance (2–4). Not only that, innate immune cells, particularly liver-resident macrophages known as Kupffer cells (KCs), work together with acquired immune cells to eliminate common pathogen-associated molecular patterns (PAMPs) and damage-associated molecular patterns (DAMPs) in the body. Besides, KCs also have a crucial role in maintaining liver homeostasis by engaging in phagocytosis, eliminating dead and senescent cells, and promoting tissue repair (5–7). In addition, the liver has the highest concentration of macrophages, which are dispersed throughout a network of circulatory channels that can quickly detect pathogens in the hepatic portal system (8).

**Figure 1:**
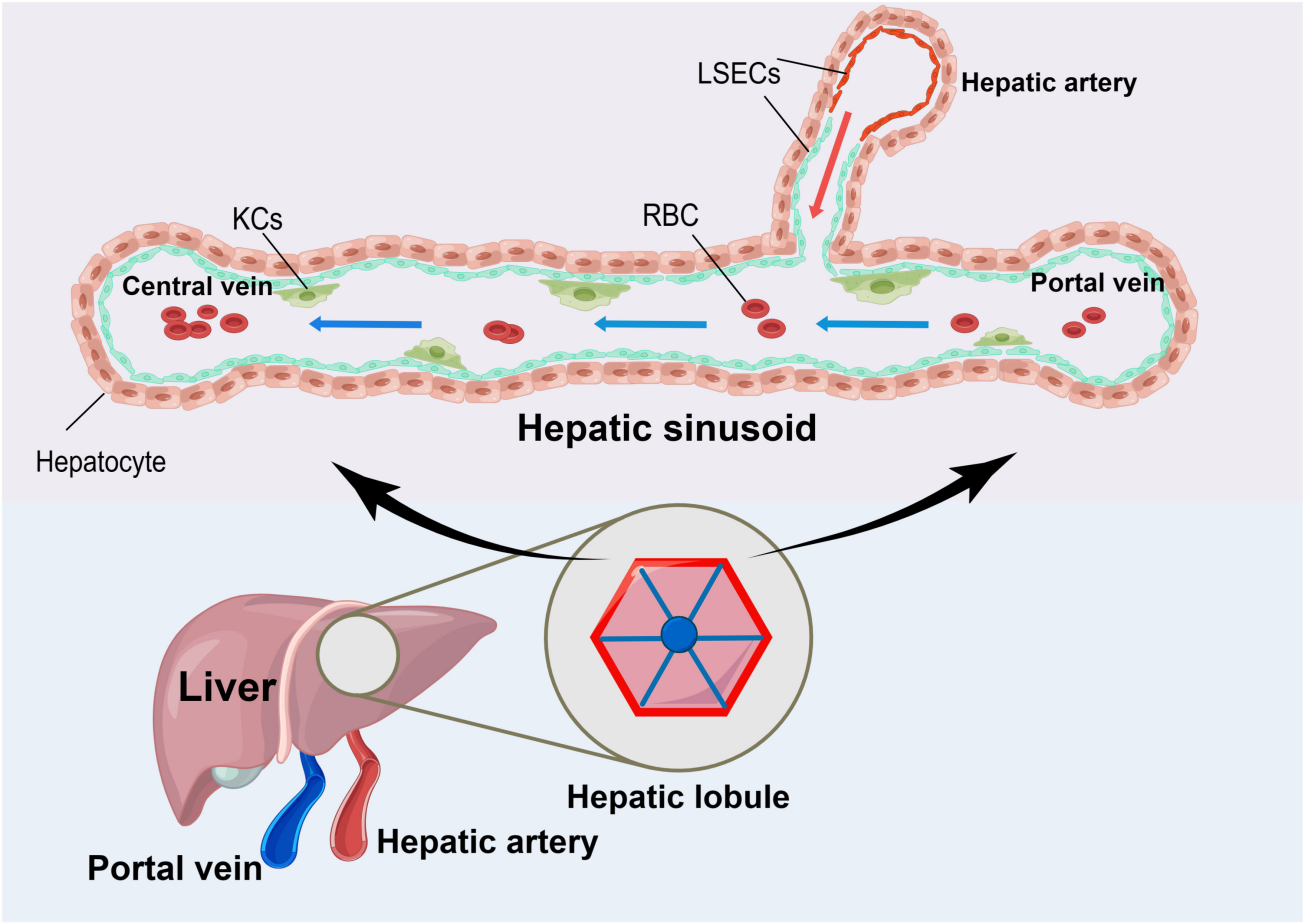
The Unique Anatomy of the Liver. The liver has a unique anatomy in that it receives blood from both the hepatic artery and the portal vein. Both sets of blood supplies end in liver sinusoidal endothelial cells (LSEC) with a large number of Kupffer cells (KCs) attached to their surface. When the blood flows through this area, it moves slowly in sinusoidal waves at a slow pace. This allows for the effective absorption of nutrients and nourishment of the liver tissues. Additionally, it enables the attached KCs to remove disease-causing substances, thereby maintaining the body’s homeostasis.

The liver has a unique anatomy because it receives blood from both the hepatic artery and portal vein. This dual blood supply nourishes to the diverse structures and cells within the liver (8). The hepatic arterial and portal circulation terminate in the liver sinusoidal endothelial cells (LSECs), which consist of a thin, porous network of special capillaries and complement KCs in the hepatic sinusoids to form a solid surveillance system (9, 10)(**Figure 1**). The blood flows in sinusoidal waves at a slow pace, which allows for prolonged exposure to antigens within the sinusoids. This facilitates the recognition and handling of antigens by both immune and non-immune cells. The portal vein supplies the liver with the majority of its blood supply (9). In addition to being rich in nutrients, the portal vein is also rich in pathogenic molecules such as lipopolysaccharide (LPS) (11). When intestinal epithelial damage or failure, it can lead to the entry of infections into the bloodstream. Then, these infections can travel from the portal vein to the liver, bypassing conventional immune organs such as the spleen and lymph nodes (8, 11, 12).

Meanwhile, the liver conducts circulating immune surveillance by mobilizing immune cells (e.g., KCs) within the liver to eliminate pathogens or toxins and maintain immune homeostasis in the liver and throughout the body. A study found that KCs crawling within the hepatic vascular system were able to effectively capture blood-borne disseminating *Borrelia burgdorferi*, thus creating an efficient surveillance and filtering system. Additionally, KCs can induce the formation of chemokine receptor (CXCR)3-dependent clusters of activated invariant natural killer T cells (*i*NKT cells) after ingesting *Borrelia burgdorferi.* This clustering prevents the spread of pathogens to organs, such as the joints (13). Therefore, KCs in the liver are considered the body’s first line of defense against any pathogens that are transmitted (8). The effect was similarly demonstrated in animal experiments by depleting KCs following intravenous administration of liposome-entrapped clodronate or using CRISPR/Cas9-mediated genome editing to prepare lacking liver immune receptor models. It has been discovered that in a mouse model, the depletion of KCs or immune receptors in the liver leads to 100% mortality from a sub-lethal dose of *Listeria* monocytogenes. However, removing the spleen did not have any impact on host immunity or survival (8, 14, 15). In short, this suggests that the liver plays a crucial role in detecting pathogens and defending the host. When the function or structure of the liver is compromised, especially for KCs, it becomes vulnerable to systemic diseases and can cause damage to multiple organs, resulting in disruptions to the immune microenvironment.

The main pathophysiological features of acute liver failure (ALF) are massive hepatocyte death and immune-inflammatory response (16). Among these, immune-mediated liver injury starts early in ALF and is primarily caused by innate immunity followed by an adaptive immune response that leads to further injury. The early activation of the innate immune system is specific to the activating substances, such as PAMPs and DAMPs. PAMPs play a more significant role in ALF induced by liver pathogens, while DAMPs are released from damaged cells and are crucial in ALF caused by hepatotoxic substances (17, 18). Monocytes and macrophages are essential components of the innate and adaptive immune response because they possess receptors on their cell surface that recognize PAMPs and DAMPs. Upon activation, they can modulate the immune response by producing reactive oxygen radicals and either anti-inflammatory or pro-inflammatory cytokines (17–19). Based on the anatomy of the liver, KCs are located in the hepatic blood sinusoids as an important defense device within the liver. They have a scavenging and filtering effect on the incoming and outgoing blood, effectively identifying toxic substances or pathogens in the blood (20, 21). In the diseased state, mononuclear macrophages recruited from outside the liver differentiate into various subsets of macrophage, leading to different functions. This process, known as polarization, determines changes in the local hepatic immune microenvironment and even the systemic immune state, especially in cases of ALF (11, 22).

Another major cause of damage in ALF is the destruction of hepatocytes by toxic substances and their death. In addition to necrosis and apoptosis, which are the accepted modes of cell death, several new modes of cell death have been identified and confirmed over the past decade. These include pyroptosis, necroptosis, and ferroptosis, which are available for study (16, 23–25). Pyroptosis is a newly discovered form of programmed cell death that specifically targets the innate immune defenses of intracellular bacteria. It plays a crucial role in defending against pathogens and danger signals (26, 27). However, excessive pyroptosis can lead to the development of ALF, as demonstrated in LPS/D-galactosamine(D-GalN)-induced ALF mice models (28). Therefore, inhibiting cellular pyroptosis has also been used as a research hotspot for ALF treatment in recent years.

At the same time, there has been interest in the relationship between cell death and the immune-inflammatory response. In ALF, dying hepatocytes release DAMPs that bind to evolutionarily conserved pattern recognition receptors of the innate immune system. These receptors are found in both liver-resident cells (e.g., KCs, LSECs) and cells that are recruited in response to injury (e.g., monocytes, macrophages, NK cells). This binding triggers the release of inflammatory mediators, including cytokines and chemokines. In turn, inflammatory mediators lead to further cell death, establishing a highly hepatotoxic feedforward cycle of inflammation and cell death (29). As important intrinsic immune cells, macrophages and their polarization play a crucial role in this process. However, there is a lack of clear description and discussion regarding the novel cell death modality known as pyroptosis. Therefore, this paper will discuss the roles and molecular mechanisms of macrophage polarization and hepatocyte pyroptosis in ALF. It will further explore how these processes alter the immune microenvironment of the liver, leading to immune dysfunction in the liver or even the entire circulatory system. Additionally, this paper will delve into the crosstalk between macrophage polarization and hepatocyte pyroptosis to provide a new theoretical basis for the pathogenesis and immunotherapy of ALF in the future.

## 2 Macrophages

### 2.1 Macrophages in normal liver

As mentioned before, the liver is supplied by two blood supply systems: the hepatic artery and the portal vein. It has immune regulation and circulatory immune monitoring functions. The portal vein is rich in both nutrients and molecules that can cause diseases, such as LPS (11). The circulating blood flows through the portal system and directly into the liver, where it undergoes a detoxifying and removal process (30). The process is primarily carried out by immune cells in the liver, with hepatic macrophages playing a crucial role (21).

The source of macrophages in a healthy liver mainly consists of self-renewing tissue-resident macrophages, such as KCs. These cells are located in the hepatic sinusoids and account for 80% of systemic macrophages (22). They also make up 20-25% of non-parenchymal cells in the liver and are the largest population of innate immune cells in the liver. KCs maintain hepatic homeostasis by removing pathogens through the portal vein (31). This regulatory immune and clearance function plays a vital role in maintaining liver function and immune system balance. Firstly, the immunomodulatory function of macrophages is to regulate both innate and acquired immune responses by releasing pro- and anti-inflammatory mediators. This helps to maintain immune balance in the body. Secondly, macrophages are also able to function as antigen-presenting cells and regulate the adaptive immune response (32). Thirdly, macrophages also function to clear harmful substances from the blood and prevent infection. This includes the clearance of translocated gut microbiota (20). Particularly, KCs are capable of specifically phagocytosing particulate material larger than 200 nm (20, 33).

But, under normal circumstances, the intestinal flora also releases some LPS into the bloodstream. So, how does the liver maintain immune tolerance without causing local inflammation? This issue is closely intertwined with the LSECs to which KCs are attached. LSECs mainly form highly permeable capillaries without basement membranes in the hepatic sinusoids. They share similar functions with KCs as antigen-presenting cells and are involved in the process of phagocytosis. LSECs and KCs collaborate to phagocytose blood-borne pathogens and substances present in the hepatic arteries and portal veins, preventing their further systemic circulation and averting widespread inflammatory reactions. However, it is important to note that LSECs have a higher responsiveness to LPS compared to KCs. This is predominantly due to the presence of Toll-like receptor 4(TLR4) and cluster of differentiation 14 (CD14) on the surface of LSECs, which enables them to directly interact with LPS, a byproduct of bacterial degradation. This interaction triggers a decrease in the expression of CD54 molecules on the surface of LSECs, which in turn reduces the adherence of leukocytes to LSECs (34). Ultimately, this leads to a decrease in localized inflammatory responses and promotes immune tolerance. In addition, LSECs bind LPS and produce prostaglandins, including prostaglandin E_2_, which can inhibit downstream gene expression induced by TLR4 ligands through nuclear receptors (35). This mechanism promotes immunological tolerance to LPS in normal conditions by inhibiting leukocyte adhesion and local activation, thereby maintaining the integrity of the liver endothelial cell layer.

The regulation of LPS tolerance by LSECs is very delicate. Because LSECs can initially tolerate LPS at a certain concentration, but as the LPS concentration gradually increases, LSECs can overcome their initial tolerance and simultaneously activate KCs. This not only ensures immune tolerance within the physiological range, but also enables an accurate response to bacterial infection during this period (36). Mechanistically, when the concentration of LPS is too high, LSECs instead increase the expression level of CD54 on the surface. This leads to an increase in leukocyte adhesion and aggregation, facilitating the local clearance of toxic substances against pathogens. Meanwhile, it has been found that LSECs can directly respond to LPS stimulation in an inflammatory environment by altering the expression pattern of their chemokine genes, such as C-C motif chemokine ligand 2(CCL2), CCL3, CCL4, and CCL7 (37, 38). Specifically, CCL2 plays a role in recruiting inflammatory monocytes into the liver (37). Therefore, LSECs and KCs play a crucial role in maintaining local immune tolerance in the liver. They have a significant regulatory function in accurately detecting high levels of LPS and facilitating the recruitment of monocytes to enhance the initial signaling, thereby initiating the downstream inflammatory cascade response.

### 2.2 Macrophages in ALF

When a liver injury occurs, the macrophage population in the liver undergoes changes. This process is primarily caused by the release of pro-inflammatory mediators or chemokines from activated KCs into the bloodstream. This triggers the accumulation of peritoneal macrophages and monocyte-derived macrophages (MoMFs) in the liver and is implicated in the development of various liver diseases (21, 22, 39).

In the pathogenesis of ALF, hepatocytes are exposed to foreign toxic substances such as acetaminophen (APAP), pathogens, and LPS produced by bacteria, which can lead to a significant number of hepatocyte deaths in the liver (40, 41). The disease progression leads to the regeneration and repair of hepatocytes, but they may not be able to fully compensate for the damage caused by cell death. After that, the dead cells release DAMPs (39), which can bind to pattern-recognition receptors (PRRs) such as Toll-like receptors (TLRs), cytoplasmic Nod-like receptors (NLRs), Retinoic acid-inducible gene (RIG)-I-like receptors (RLRs), and C-type lectin receptors (CLRs) (42). PRRs are expressed on the surface of immune cells and upon binding cause the immune cells to transform their phenotype and become activated (43), which initiates an inflammatory response. This ultimately leads to changes in the immune microenvironment in the liver (44).

As the predominant intrinsic immune cells in the liver, activated KCs release inflammatory mediators and chemokines into the bloodstream, which recruit bone marrow-derived monocytes to develop into mature MoMFs. However, in the early stages of ALF, the liver-resident KCs are gradually depleted (22, 45–48). Recent research has found that the acute injury model induced by carbon tetrachloride (CCl4) in mice has three distinct phases: necroinflammation, early repair, and late repair. At the gene and protein level, the immune microenvironment of the liver was characterized by MoMFs-induced immune damage, with lower levels of KCs observed during the necroinflammation. This finding indicates a potential role for MoMFs in the phagocytosis of necrotic hepatocytes. However, the opposite cellular distribution was observed during the repair (48). Therefore, the majority of macrophages in ALF are replaced by MoMFs, which can perform either pro-inflammatory or anti-inflammatory functions.

The pro-inflammatory and anti-inflammatory effects described here are illustrated in two main ways. To begin with, the pro-inflammatory effect of MoMFs is due to their high expression of the C-C motif chemokine receptor 2(CCR2) and surface marker Ly-6C (CCR2^+^Ly-6C^high^ MoMFs). Their main function is to clear toxic substances from the liver by releasing vasoactive and inflammatory mediators such as Term1 and S100 calcium binding protein A8 and A9 (S100A8/9) into the peripheral blood during the early phase of acute liver injury (11, 49). S100A8/9 is a novel molecule of DAMPs that can bind to TLR4 receptors, promoting inflammation propagation and activating other relevant immune cells (50, 51). During the disease’s repair phase, MoMFs and yolk sac-derived KCs undergo a transformation into anti-inflammatory MoMFs after being stimulated by macrophage-colony stimulating factor 1 (CSF1) (52, 53). The phenotypic transition from pro-inflammatory CCR2^+^Ly-6C^high^ MoMFs to anti-inflammatory CCR2^-^Ly-6C^low^ MoMFs, which secrete anti-inflammatory factors, facilitate hepatic repair, suppresses inflammation, and maintain the stability of the hepatic immune microenvironment (11, 22, 49, 54). In addition, the release of anti-inflammatory factors from CCR2^-^Ly-6C^low^ MoMFs into the bloodstream contributes to the deactivation of functional monocytes and increases the risk of sepsis (22, 54). Briefly, it was observed that MoMFs had the ability to undergo differentiation towards either M1 macrophages, representing a classic proinflammatory phenotype, or M2 macrophages, representing an alternative anti-inflammatory phenotype in different phases of the disease (55, 56).

However, monocytes and other immune cells are recruited to the liver from the systemic circulation, resulting in a relative decrease in the number of immune cells and immunity in the systemic circulation. This can lead to an increased risk of systemic opportunistic infections (45, 57). In particular, the occurrence of bacterial translocation in the gut releases PAMPs, which can easily initiate systemic infections (43, 58) and enhances hepatocyte death by binding to TLRs (59). In conjunction with macrophage-derived mediators, they can also cause vascular endothelial dysfunction and microcirculatory disturbances. These disturbances can result in extrahepatic organ dysfunction (22), which is part of a larger process known as systemic inflammatory response syndrome (SIRS). If left untreated, SIRS can progress to sepsis, septicemia, or even multi-organ failure, ultimately leading to a poor prognosis for patients with ALF (60–62).

Therefore, there is a conflict regarding the role of macrophages in ALF. Some studies suggest that recruited monocytes develop into mature macrophages with an improved ability to clear hepatotoxic substances and alleviate liver damage. At the same time, some scholars believe that the pro-inflammatory capacity of macrophages in ALF will further exacerbate liver damage and induce SIRS. Moreover, simply eliminating or impairing the function of various immune cells will unavoidably cause a delay in the healing process of damaged tissue. This underscores the crucial role of the immune system in tissue repair. In general, liver macrophages cannot be restricted to a single role. Their phenotypes can change according to the altered immune microenvironment in the liver, and they perform different functions both in the liver and systemically to maintain the balance of the immune microenvironment. Such conflicting and mutually limiting roles also pose one of the main challenges in the development of ALF immunotherapies. This is because potential molecular targets may have varying local and systemic effects (22, 32, 54).

## 3 Macrophage polarization

**Figure 2:**
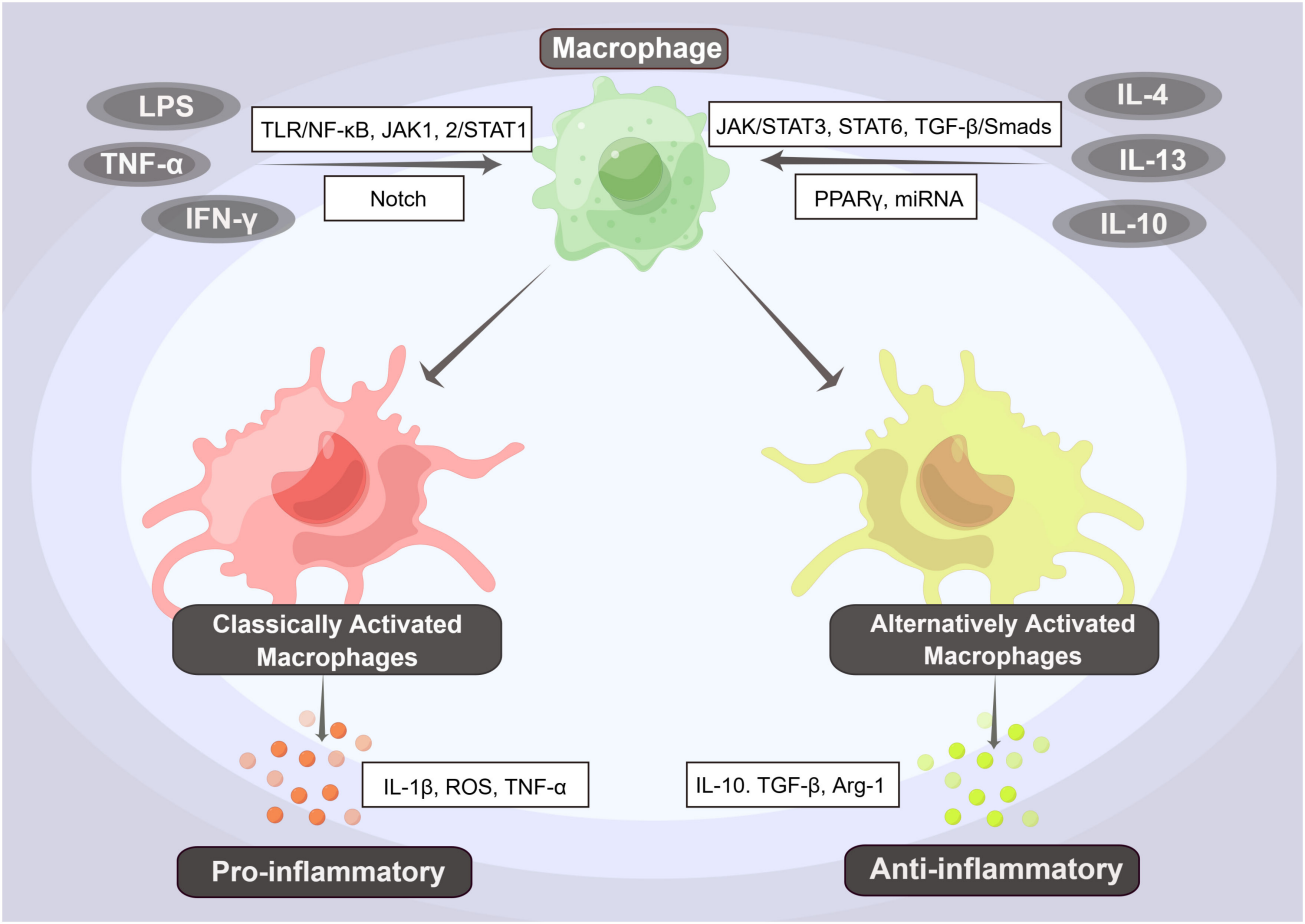
The Phenotypes and Pathways of Macrophage Polarization. Primary macrophages can differentiate into pro-inflammatory (M1) and anti-inflammatory (M2) macrophages by activating various factors and pathways. Among them, factors such as LPS, interferon-γ (IFN-γ), and tumor necrosis factor-α (TNF-α) can induce the differentiation of primitive macrophages into M1-type macrophages, i.e., classically activated macrophages (CAMs), through activation of the TLR/NF-κB, JAK1,2/STAT1, and Notch signaling pathways. These M1-type macrophages produce a large number of pro-inflammatory factors, such as interleukin (IL)-1β, reactive oxygen species (ROS), and TNF-α, further inducing a proinflammatory response. In contrast, when anti-inflammatory factors such as IL-4, IL-13, and IL-10 activate the JAK/STAT3, STAT6, and TGF-β/Smads signaling pathways, they induce the differentiation of M2-type macrophages, also known as alternatively activated macrophages (AAMs). These macrophages produce anti-inflammatory factors such as IL-10, TGF-β, and Arg-1, which initiate an anti-inflammatory response.

In addition to regulating the immune system and performing phagocytosis, macrophages are also highly diverse and adaptable. They can exhibit various functions depending on the stimuli or proteins present in the immune microenvironment and can differentiate into different subtypes through a process known as polarization (63–67). Macrophages can be classified into two phenotypes: pro-inflammatory (M1) and anti-inflammatory (M2). These phenotypes are determined by various factors, including microorganisms, tissue microenvironment, and cytokine signals (64, 68, 69). M1 macrophages are induced by various stimuli, including LPS, interferon-γ (IFN-γ), and tumor necrosis factor-α (TNF-α), which are Th1 cytokines. Additionally, inducible nitric oxide synthase (iNOS) and granulocyte-macrophage colony-stimulating factor (GM-CSF) can also induce M1 macrophages (31, 70). Primitive macrophages differentiate into M1 macrophages, which produce a large number of pro-inflammatory factors, such as interleukin (IL)-1β, reactive oxygen species (ROS), and TNF-α. These factors mediate antimicrobial defense, tissue destruction, and antitumor resistance (31, 70, 71). In contrast, M2 macrophages are induced by anti-inflammatory factors such as IL-4, IL-13 (which are Th2 cytokines), IL-10, and transforming growth factor-β (TGF-β). These macrophages produce anti-inflammatory factors. M2 macrophages are primarily involved in wound repair, angiogenesis, resistance to parasites, and promotion of tumor growth (67, 70–72). The underlying mechanisms are even more complex, involving multiple signaling pathways and associated regulatory factors (**Figure 2**).

### 3.1 M1 macrophage

**Figure 3:**
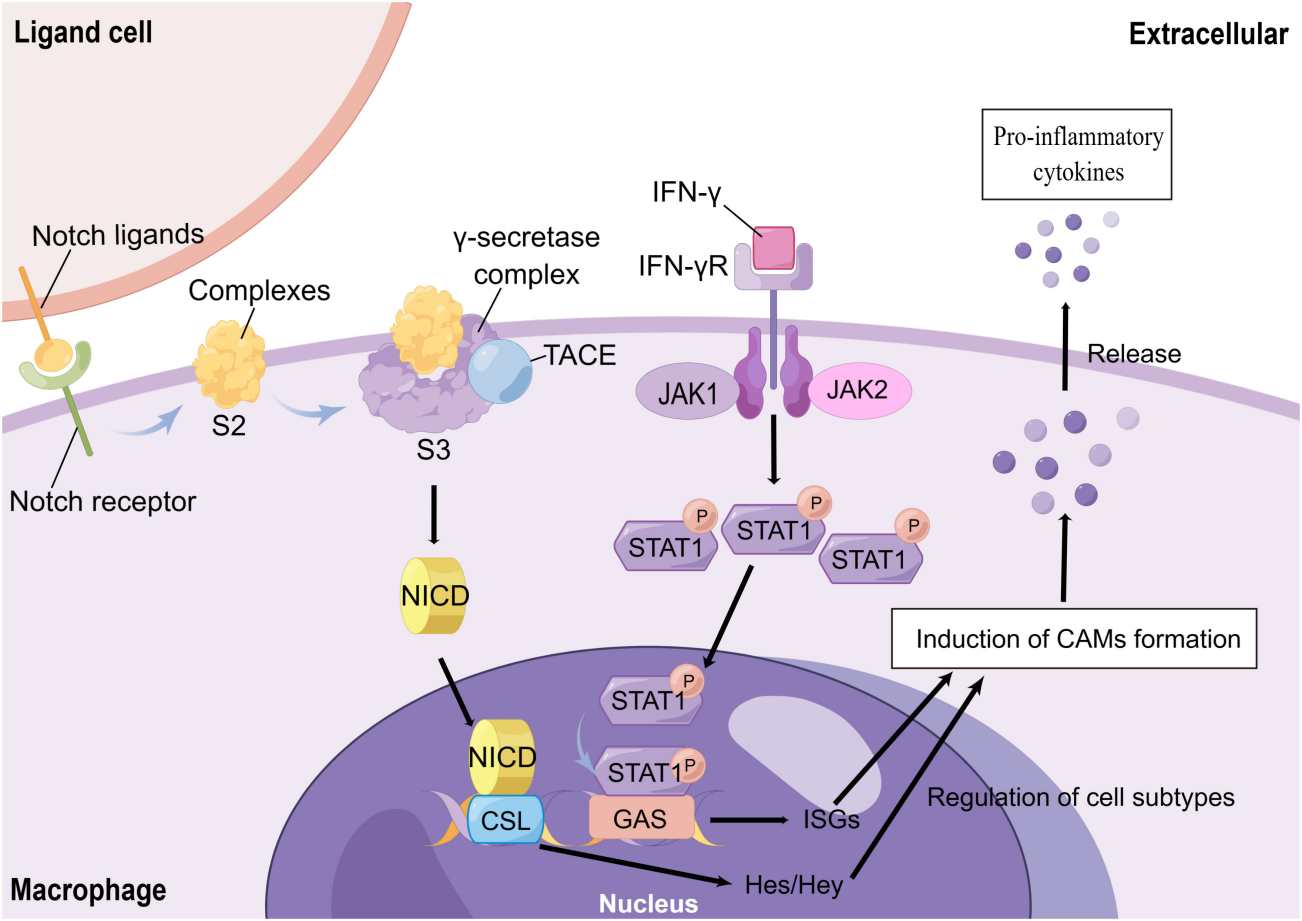
The Mechanisms of CAMs Formation. Notch receptors, which are expressed on the surface of macrophages, bind to neighboring cellular ligands. The ligand-receptor complex is formed and then exposed to hydrolysis site 2 (S2) in the extracellular proximal membrane region after endocytosis. After cleavage by tumor necrosis factor-α-converting enzyme (TACE) and hydrolysis of the γ-secretase complex (located at site S3 in the transmembrane region), a soluble Notch intracellular domain (NICD) is formed. This NICD then enters the cytoplasm and translocates to the nucleus, where it binds to the nuclear CSL transcription factor complex. This binding further activates Hes and Hey, induces the formation of CAMs, and mediates the release of inflammatory factors. On the other hand, the interferon-γ (IFN-γ) receptor (IFN-γR) on the surface of macrophages activates JAK1/JAK2 upon IFN-γ stimulation, which leads to the phosphorylation of intracellular STAT1. Phosphorylated STAT1 then enters the nucleus and binds to the Gamma interferon activation site (GAS) DNA element. This binding induces the transcription of interferon-stimulated genes (ISGs) and triggers the formation of CAMs.

M1 macrophages, also known as classically activated macrophages (CAMs), are characterized by the release of large amounts of inflammatory cytokines, Th1 chemokines, and ROS/RNS products. They also act as positive feedback to unpolarized macrophages (70, 73, 74). The regulation of M1 polarization is primarily controlled by the TLR/nuclear factor-kB (NF-kB) signaling pathway, the Janus kinase 1, 2 (JAK1, 2)/signal transducer and activator of transcription 1 (STAT1), and the Notch signaling pathway. LPS is the main factor for the activation of the TLR/NF-κB signaling pathway (75). It promotes the polarization of CAMs by binding to TLR4 receptors on the surface of initial macrophages and activating NF-κB via the MyD88-dependent pathway or interferon regulatory factor 3 (IRF3). This activation leads to the production of IL-6 and iNOS (76, 77), while the level of IL-10 decreases. Ultimately, this process mediates the formation of the pro-inflammatory phenotype of CAMs (71, 78, 79). Activation of NF-κB p65 is a marker of CAM activation (79). However, the binding of IFN-γ to its receptor (IFN-γR) activates JAK1 and JAK2, which are members of the tyrosine kinase family. This activation leads to the phosphorylation of STAT1, which then translocates into the nucleus to bind the conserved Gamma interferon activation site (GAS) DNA element. This binding activates the transcription of interferon-stimulated genes (ISGs), resulting in the formation of CAMs and the promotion of chemokine and antigen-presenting molecule production (80, 81) (**Figure 3**). Moreover, the JAK1,2/STAT1 signaling pathway and TLR/NF-κB signaling pathway have synergistic effects (67, 81, 82).

Recently, the Notch signaling pathway has also received widespread attention in the polarization of CAMs. Macrophages stably express Notch ligands and Notch 1, 2, and 4 receptors on their surface, which can bind to ligands or receptors in adjacent cells. On the ligand cells, endocytosis of the ligand-receptor complexes leads to a change in the mechanical conformation of the endocytosed receptor. This change exposes the complexes to the hydrolysis site 2 (S2) in the extracellular near-membrane region. The complexes are cleaved by tumor necrosis factor-α-converting enzyme (TACE) and hydrolyzed by the γ-secretase complex (located at the S3 site in the transmembrane region). This process forms a soluble Notch intracellular domain (NICD) that enters the cytoplasm and translocates to the nucleus. NICD binds to the nuclear CSL transcription factor complex and activates the hairy enhancer of split (Hes) and Hes with YRPW motif (Hey) family members, which are classical Notch target genes. These genes induce CAMs and mediate the release of inflammatory factors (**Figure 3**). On the other hand, blocking Notch signaling could promote the polarization of M2 cells (66, 83–85). Not only that, but the Notch pathway regulates a variety of biological properties of macrophages that still need further exploration.

### 3.2 M2 macrophage

M2 macrophages are also known as alternatively activated macrophages (AAMs). AAMs are characterized by the production of large amounts of anti-inflammatory cytokines, Th2 chemokines, C-type lectins, clearance receptors, and polyamines (66, 78). It is mainly regulated by signaling pathways such as JAK/STAT3, STAT6, TGF-β/Smads, peroxisome proliferator-activated receptor γ (PPARγ), and certain miRNAs. Th2 cytokines IL-4 and IL-13 inhibit M1 polarization and promote AAM formation mainly through the corresponding receptors (IL-4Rα), which ultimately activate STAT6. Furthermore, STAT3 is equally important for the formation of AAMs, in addition to IL-4 and IL-13, other cytokines such as IL-10 are also activated (67, 78, 86, 87). Similarly, TGF-β induces phosphorylation of type I receptors by binding to type II receptors on the macrophage surface. This leads to the activation of Smad2 and Smad3 (88), which promotes the formation of AAMs and suppresses CAMs (67, 89). PPARγ is an essential transcription factor for cell differentiation (67, 90). It coordinates M1/M2 cell homeostasis with NF-κB and promotes the polarization of M2 cells (91). Activation of NF-κB p50 is critically important for the polarization of AAMs *in vitro* and *in vivo* (79). In recent years, the development of stem cell transplantation technology has raised significant interest in the immunomodulatory role of stem cells in various diseases, especially in the regulation of immune cells. Stem cells primarily exert their effects through the release of exosomes. It contains a large number of functional microRNAs (miRNAs) that regulate M1 and M2 macrophage polarization by targeting various transcription factors (74, 92, 93). Besides the classical signaling pathways mentioned above, the Wnt/β-catenin pathway and the PI3K/Akt/mTOR pathway have been demonstrated to have a significant impact on the regulation of macrophage polarization (94, 95). Nevertheless, M2 macrophage typing can be refined and further subdivided into M2a, M2b, M2c, and M2d subtypes, depending on their specific function and the genes they express (96).

### 3.3 Macrophage polarization in ALF

The pathogenesis of ALF is complex and involves interactions between pathogenic agents and the host immune system. This interaction leads to the disorganization of the hepatic immune microenvironment and the simultaneous apoptosis of hepatocytes. Therefore, polarization is a dynamic process in ALF, as the polarized CAMs and AAMs still retain their plasticity and can interconvert again depending on the changing environment (22, 46). In the early stage of ALF, liver damage predominates due to immune injury. When the liver is invaded by foreign toxins or bacteria, or exposed to hepatotoxic substances, it can result in the death of hepatocytes. This, in turn, triggers the release of PAMPs by pathogens and DAMPs by dying hepatocytes. These PAMPs and DAMPs activate macrophages by binding to PR receptors (e.g., TLRs, NLRs) on the surface of macrophages. This activation prompts macrophages to shift towards the M1 pro-inflammatory phenotype and release inflammatory mediators into the bloodstream. Consequently, this process recruits and activates numerous inflammatory cells in the liver in order to eliminate pathogenic bacteria from the liver (97). However, simultaneously, it leads to a substantial infiltration of inflammatory cells and the formation of an excessive release of cytokines in the liver, initiating a “cascade activation” that results in a detrimental cycle (22). This phenomenon has also been observed during the early stages of injury in the model of ALF induced by APAP (31). Such an excessive inflammatory response not only exacerbates liver necrosis and injury but also increases the risk of extrahepatic and systemic infections. Hence, during the middle and late stages of ALF, the immune system of the body is functionally suppressed as a consequence of macrophages being excessively activated. This in turn leads to the suppression of both the functions of presenting antigens and pro-inflammatory functions. Consequently, a state of functional depletion arises. Both intrahepatic and extrahepatic immune components exhibit signs of immune paralysis (98). Furthermore, the remaining macrophages undergo a shift in function from the inflammatory M1 phenotype to the anti-inflammatory M2 phenotype, which facilitates tissue repair (99, 100). This is an antagonistic effect of the body aimed at protecting against an early, excessive inflammatory response (101). However, as a result of premature over-activation and depletion, the immune cells and their function become compromised, elevating the vulnerability to opportunistic infections. Consequently, the immune function of the body is further weakened. It is important to note that the direction of macrophage polarization during ALF is not absolute and relies on the influence of various cytokines and mediators in the immune microenvironment on primitive macrophages (99). Therefore, macrophage M1/M2 regulation can significantly modulate the systemic immune microenvironment and initiate a cascade of immune responses.

The imbalance of M1/M2 macrophage polarization is a key factor in the pathogenesis of ALF and plays a central role in the imbalance of the immune microenvironment in ALF (102). In the thioacetamide (TAA)-induced acute liver injury (TAA-ALI) mice model, the expression of senescence-associated secretory phenotype (SASP) was significantly increased, inducing M1 macrophage polarization. This exacerbates liver injury in ALI through repression of autophagy-related gene 5 (ATG5) (103). Similarly, the CC-5013, a TNF-α inhibitor, was able to significantly ameliorate liver damage in ALF by reducing the proportion of CAMs through the inhibition of the TNF-α/HMGB1 signaling pathway (104). Overall, CAMs have a positive effect on liver injury, making them a potential strategy for ALF treatment. Upregulating STAT6 by mesenchymal stem cells (MSCs) can increase the proportion of AAMs and significantly alleviate liver injury in a study, which demonstrates a positive therapeutic effect in ALF (105). Similarly, the overexpression of hepatocyte nuclear factor 4α (HNF4α) increased the transcription of IL-10 and promoted the polarization of AAMs through the IL-10/STAT3 pathway. This novel therapy for ALF resulted in the alleviation of ALF (106). In the D-GalN/LPS-induced ALF mice model, treatment with JWH-13, a cannabinoid receptor 2 (CB2) agonist, attenuated alanine aminotransferase (ALT) levels and reduced the production of pro-inflammatory cytokines, thereby protecting against ALF-associated death. Not only that, pretreatment of macrophages *in vitro* with JWH-133 significantly increased the secretion of the anti-inflammatory cytokine IL-10 in CAMs. It also enhanced the expression of AAMs markers, such as Arg and IL-10. These findings suggest that JWH-133 promotes the transformation of M1 to M2 macrophage phenotype, thereby improving ALF (107). At the same time, exosomes derived from human umbilical cord MSCs inhibited macrophage activation and the production of inflammatory cytokines *in vitro* and *in vivo* when exposed to LPS. This was achieved by releasing miR-455-3p, which resulted in reduced levels of serum inflammatory factors and improved IL-6-induced acute liver injury in ALF (108). In addition, stem cells can also regulate the direction of macrophage polarization by releasing cytokines (74). A study reported that treatment with MSCs in a D-GalN-induced ALF model induces the MSCs to secrete IL-4 in a paracrine manner. This secretion promotes the phenotypic conversion of inflammatory CAMs to anti-inflammatory AAMs, leading to improved ALF (109). Therefore, adjusting the proportion of M1/M2 macrophages has become a hot topic in ALF therapy. However, it also presents a new challenge for clinical application, as CAMs are essential cells for the clearance of toxic substances in the liver. The timing of their application still requires further experimental validation. This validation should consider the benefits and adverse consequences of reducing initial immune activation and its harmful downstream effects.

## 4 Pyroptosis

### 4.1 The mechanisms of pyroptosis

Pyroptosis, as a novel mode of cell death, has received much attention in recent years, and its specific mechanisms have been well explained (26). There are two activation pathways for pyroptosis: the canonical pathway, which depends on Caspase-1, and the non-canonical pathway, which depends on Caspase-4/5/11 activations (26, 27). When damage mediators enter the tissue, they induce the release of pro-inflammatory factors and the activation of immune cells, further stimulating the formation of the intracellular inflammasome, which can be found in various cells, such as macrophages, neutrophils, and hepatocytes (110). Inflammasomes, intracellular multiprotein complexes, consist of three parts: a cytosolic sensor, a bridging adaptor, and an effector (27, 111). The cytosolic sensor of the inflammasome is formed by nucleotide-binding oligomerization domain NLRs, with NLRP3 predominantly mediating pyroptosis. Apoptosis-associated speck-like proteins containing caspase recruitment domains (ASCs) act as bridging junctions for the inflammasome, with pro-caspase-1 serving as the effector. Therefore, NLPR3 and pro-caspase-1 form an inflammasome by binding to ASCs (27, 112, 113). The canonical pathway is initiated by the recognition of different endogenous and exogenous damage factors, such as DAMPs and PAMPs, by the inflammasome. This recognition triggers the activation pro-caspase-1, leading to its maturation into caspase-1, the effector molecule. It is released into the cytoplasm act on the NF-κB signaling pathway and promotes the cleavage of pro-IL-1β and pro-IL-18 into mature cytokines (27, 113). Activated Caspase-1 cleaves the gasdermin D (GSDMD) protein into N- and C-terminal fragments (114, 115). The GSDMD-N-terminal protein folds on the cell surface, forming a membrane pore. This pore allows for the release of cell contents, including IL-1β and IL-18 pro-inflammatory cytokines, outside the cell. As a result, the cell becomes swollen and highly permeable to the plasma membrane due to an imbalance of intra- and extracellular fluids. Eventually, the cell rapidly lyses, a process known as pyroptosis (27, 114–116).

**Figure 4:**
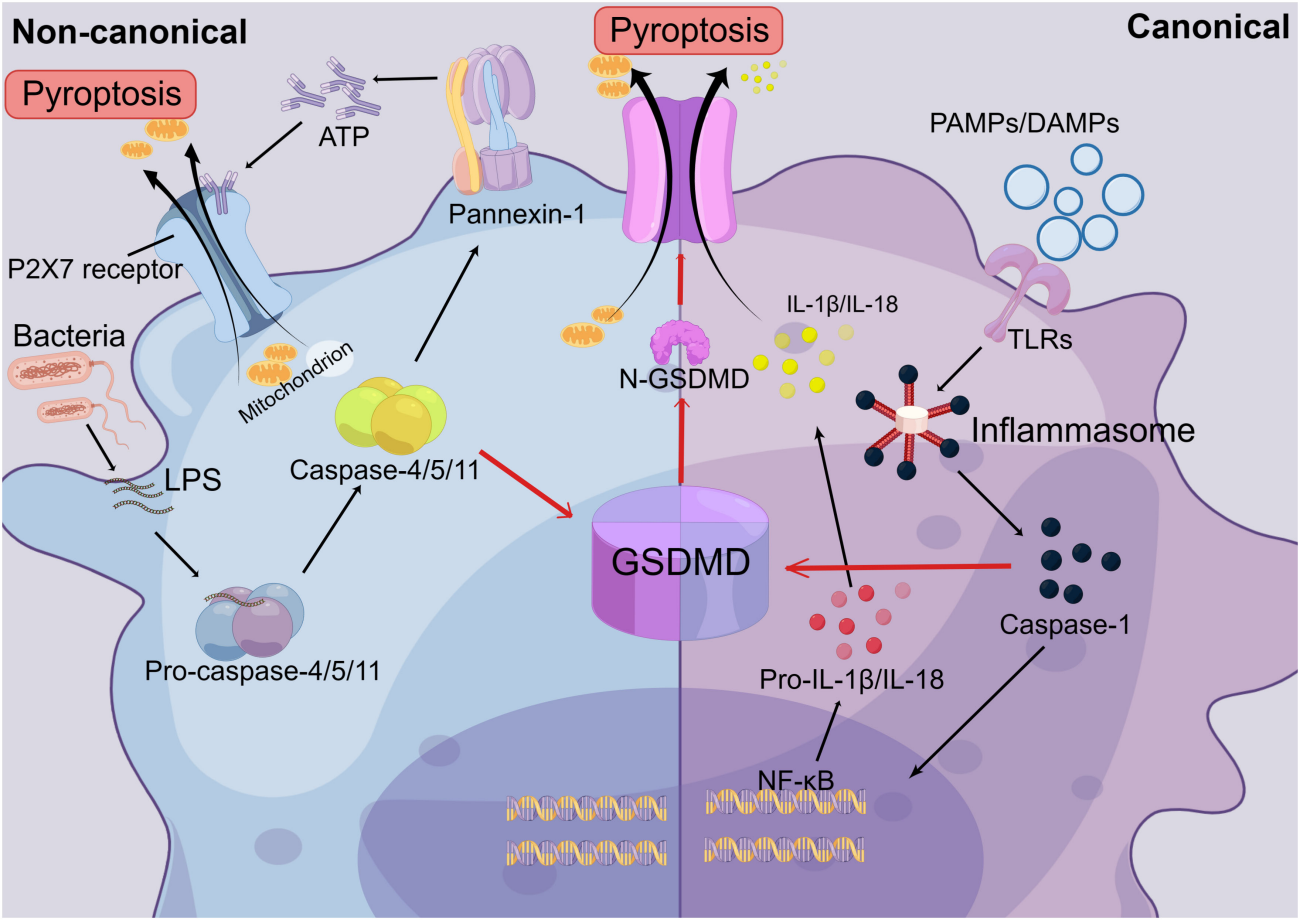
The Mechanisms of Pyroptosis. In the canonical pathway of pyroptosis, Toll-like receptors (TLRs) recognize extracellular pathogen-associated molecular patterns (PAMPs) and damage-associated molecular patterns (DAMPs), leading to the formation of inflammasomes. This, in turn, activates pro-caspase-1, causing it to mature into caspase-1. Caspase-1 is then released into the cytoplasm and acts on the NF-κB signaling pathway, promoting the maturation of IL-1β and IL-18. Secondly, activated caspase-1 cleaves the gasdermin D (GSDMD) protein into N-terminal and C-terminal fragments. The N-GSDMD fragments form pore membranes on the cell surface, allowing the release of IL-1β and IL-18 from the cell, thereby triggering inflammatory responses. Cell contents can also flow out through the pore membrane, resulting in an imbalance of intracellular and extracellular fluids and rapid cell lysis. In the non-canonical pathway, extracellular LPS can directly enter the cell and bind to the CARD domain on the intracellular Caspase-4/5/11 precursor, promoting the activation of mature Caspase-4/5/11. This activation leads to the formation of the N-GSDMD pore membrane, which triggers cellular pyroptosis. Additionally, it activates the ATP membrane channel, pannexin-1, which leads to the release of ATP from the cell. ATP binds to the P2X7 receptor through autocrine or paracrine mechanisms, resulting in the opening of the P2X7 pore. This leads to the release of cellular contents and ultimately triggers pyroptosis.

In the non-canonical pathway, LPS, which is the primary stimulus in the non-classical pathway, enters the cell directly. Its Lipid A portion then binds to the CARD structural domain on the pro-Caspase-4/5/11, promoting the activation of mature Caspase-4/5/11 (117, 118). Caspase-4/5/11 not only cleaves GSDMD to form pore membranes like the canonical pathway that leads to pyroptosis, but it can also activate pannexin-1, the membrane channel for ATP, which induces the extracellular release of ATP (27). In the extracellular space, ATP binds to the P2X7 receptor through an autocrine or paracrine mechanism, causing the opening of the P2X7 pore and resulting in pyroptosis (119, 120). At the same time, Caspase-4/5/11 induces the formation of the NLRP3 inflammasome by promoting K-ion efflux and activating the classical scorch pathway (27, 119). Therefore, there is a distinction and link between the non-canonical and canonical pathways (**Figure 4**). The nature of pyroptosis is an effective immune defense against bacteria-infected cells in the internal environment. And IL-1β and IL-18, released from the cleaved cell by pyroptosis, are potent pro-inflammatory cytokines that can recruit innate immune cells to the site of infection and regulate acquired immune cells, aiding in the capture and clearance of pathogens (121, 122). This immune response towards pathogens facilitates the elimination of foreign microorganisms. However, if not well regulated, this excessive pro-inflammatory cascade response and host cell pyroptosis can be harmful to healthy tissue (27, 120, 123). Moreover, mature IL-18 can promote the production of IFN-γ and enhance the cytolytic activity of NK cells and T cells. (121, 122).

### 4.2 Pyroptosis in ALF

Inflammasome formation is also present in hepatocytes. Hepatocyte pyroptosis, induced by the activation of the specific NLRP3 inflammasome in hepatocytes, is considered to be a significant contributor to liver injury and liver fibrosis (27, 124). The production of NLRP3 inflammasome, cleavage of Caspase-1, and elevated levels of IL-1β factor have been observed in the concanavalin (ConA) and LPS/D-GalN induced liver failure models (27, 125, 126). In addition, the levels of proteins associated with pyroptosis, including caspase 1/4, GSDMD-N, IL-1β, IL-18, TNFα, and IFN-γ, were also detected in liver tissue from patients with ALF (127). Therefore, numerous studies have been conducted to alleviate liver damage in ALF by inhibiting key proteins or genes involved in pyroptosis. This is considered a potential therapeutic mechanism for treating ALF.

GSDMD, as a pyroptosis executioner (128), has been the primary focus of several studies. Using necrosulfonamide (NSA), an inhibitor of GSDMD, on the LPS/D-GalN-induced ALF mouse model resulted in a significant improvement in the pathophysiology and serology of liver damage. Additionally, it significantly decreased the levels of GSDMD, NLRP3, Caspase-1, and IL-1β (28). This study not only highlights the significance of pyroptosis in the progression of ALF but also demonstrates that inhibiting pyroptosis *in vivo* can effectively mitigate liver damage associated with ALF and yield therapeutic benefits. Similarly, limonin was able to inhibit LPS-induced pyroptosis by preventing cell membrane rupture and GSDMD activation. Additionally, limonin could prevent LPS-induced liver injury by primarily reducing the expression of NLRP3 and Caspase-1-related proteins, thereby inhibiting IL-1β (129). The 3,4-dihydroxyphenylethyl alcohol glycoside (DAG) isolated from Sargentodoxa cuneata has been shown to possess antioxidant, anti-apoptotic, and anti-inflammatory effects. Further studies have revealed that DAG reduces the levels of pyroptosis-related factors IL-1β, IL-18, and ROS. It also inhibits the expression of Caspase-1 and GSDMD in a dose-dependent manner, thereby inhibiting pyroptosis to treat APAP-induced ALF (130). The tyrosine-alanine (YA) peptide, which is a significant constituent of oyster (Crassostrea gigas) hydrolysate (OH), has demonstrated a hepatoprotective effect. It reduces the upregulation of GSDMD, the activation of caspase-1, and the cleavage of the C-terminus of GSDMD in mice injected with LPS/D-GalN (131).

Besides that, it has also been reported that PAMPs and DAMPs can directly or indirectly cause hepatocyte pyroptosis through cell-to-cell crosstalk (27). And it is not only hepatocyte pyroptosis, but macrophage pyroptosis also contributes to the development of liver disease (132, 133). In summary, pyroptosis is a promising therapeutic target for inflammatory diseases. This can be achieved by blocking related molecules such as NLRP3, Caspase-1, and GSDMD, which ultimately affects the progression of ALF. However, pyroptosis is an important defense mechanism against pathogenic invasion by its nature. Under physiological circumstances, moderate pyroptosis plays an important role in host defense against pathogenic microorganisms (134). Many pathogens have developed antimicrobial activity against macrophages, which are intrinsic immune cells. These pathogens can invade and replicate within macrophages, effectively isolating themselves from extracellular immune defenses and allowing them to escape the immune system. However, these pathogens cannot resist extracellular immunity (135). Bacteria, on the other hand, can activate the formation of various pyroptosis-associated inflammasomes, such as Listeriolysin released by *Listeria* monocytogenes, *B. anthracis* protease lethal factor (LF), Pneumolysin (PLY), and α-hemolysin released by *Staphylococcus aureus*. All of these are known to be activators of NLRP3 (136). LF was the first activator of pyroptosis to be identified and discovered. Mechanistically, LF is cleaved intracellularly and further degraded by the proteasome (137). The degradation product can then participate in the formation of CARD at the C-terminus of Caspase-1 (138, 139). This activation leads to the formation of inflammasomes and triggers the canonical pathway, inducing pyroptosis. As a result, infected cells rupture, releasing pathogens into the extracellular environment and initiating an immune response to eliminate the pathogens. In addition, the gene sequence of LPS released by Gram-negative bacteria, LipidA, is a highly conserved. LipidA binds to Caspase-11/4/5 and triggers the oligomerization and activation of caspases, leading to the induction of the non-canonical cellular pyroptosis (140, 141). Overall, this mechanism serves as a clearance mechanism for the organism to defend against invading pathogens and plays a crucial role in protecting the organism from such pathogens. Therefore, therapeutic interventions aimed at inhibiting pyroptosis may have inherent flaws. It can worsen the existing pathogenic invasion and increase the risk of opportunistic infections. Furthermore, when regulating the local signaling pathways or key proteins in the organism, it is necessary to consider the systemic response that it triggers. The pros and cons of this issue are unavoidable and will require extensive research before it can be translated into clinical treatment. It is indisputable that conducting comprehensive studies on pyroptosis is essential for understanding the pathogenesis of ALF and for the development of drugs targeting this process.

## 5 The crosstalk of pyroptosis and macrophage polarization in ALF

Macrophage polarization and pyroptosis are important for the development of ALF and share certain signaling pathways or regulatory mediators. Therefore, it is reasonable to speculate that there is an interaction between macrophage polarization and pyroptosis. However, there are fewer studies on the mutual regulation and crosstalk between macrophage polarization and pyroptosis. Cluster of Differentiation 38 (CD38) is a type II transmembrane protein that is widely expressed in immune cells. It controls the innate immune response and inflammatory pathways triggered by infection (122, 142). It was found that liver-injured mice with CD38 knockdown exhibited more severe pyroptosis and liver damage. By comparing protein expression in WT and CD38-deficient mice, researchers found elevated expression of M1 macrophage marker proteins such as TLR4, MyD88, and phosphorylated NF-κB p65 in CD38-deficient mice. Furthermore, the increased expression of pyroptosis-associated markers caused by CD38 knockdown could be reversed by TLR4 mutation. This suggests that the more severe liver damage and pyroptosis caused by CD38 deficiency are related to the TLR4 signaling pathway. However, further research is needed to elucidate the role of CD38 in M1 macrophages and pyroptosis through the TLR4 signaling pathway is not available (122). TLR receptor activation has been found to induce the production of the NLRP3 inflammasome and the development of pyroptosis (143). Therefore, we can speculate that CD38 inhibits pyroptosis by regulating the TLR4 signaling pathway. But it remains to be considered whether CD38 further regulates pyroptosis through the polarization of M1 macrophages.

High mobility group box protein 1 (HMGB1) is a nuclear DNA-binding protein that activates Caspase-1-dependent pyroptosis in hepatocytes, thereby exacerbating the inflammatory response and damage. This process can be ameliorated by HMGB1 inhibitors (144). In addition, HMGB1 also acts as a DAMP that easily translocates to the outside of cells in response to endogenous tissue damage or exogenous microbial invasion. It activates immune cells and releases pro-inflammatory factors, which cause an inflammatory response (145). When the DAMPs activate macrophages by binding to PRR on the surface of the macrophage, the activated macrophage will secrete the pro-inflammatory HMGB1 (22, 144). Once HMGB1 translocates to the outside of the cell membrane or is transported to target cells via extracellular vesicles, it binds to its receptor RAGE or TLR4 and initiates as a DAMP molecule. This leads to the activation of the NLRP3 inflammasome, inducing pyroptosis in recipient cells and provoking an inflammatory response (144, 146–148). Moreover, the HMGB1 outside the cell could activate the MyD88-dependent TLR4 signaling pathway and enhance NF-κB expression through TLR4 binding (122, 144). Therefore, macrophages following LPS induction will release HMGB1 to initiate hepatocyte pyroptosis. This process can also induce the formation of the NLRP3-inflammasome through activation of the TLR4/MyD88/NF-κB signaling pathway (144). As discussed in a previous paragraph, this pathway is also considered a classical pathway for M1 macrophage polarization. Although the role of macrophage polarization is not highlighted in this article, HMGB1 plays an important role in liver damage of ALF as a common mediator of both macrophage polarization and pyroptosis, and the therapeutic effect of HMGB1 inhibition on ALF has been demonstrated in several studies. Besides, in the LPS/D-GalN-induced ALF mouse model, it was discovered that lenalidomide (CC-5013) treatment significantly reduced the activation of the TNF-α/HMGB1 signaling pathway. This reduction resulted in a decrease in the number of M1 macrophages in both liver and kidney tissues, ultimately leading to a decrease in intra-tissue pyroptosis levels (104).

**Figure 5:**
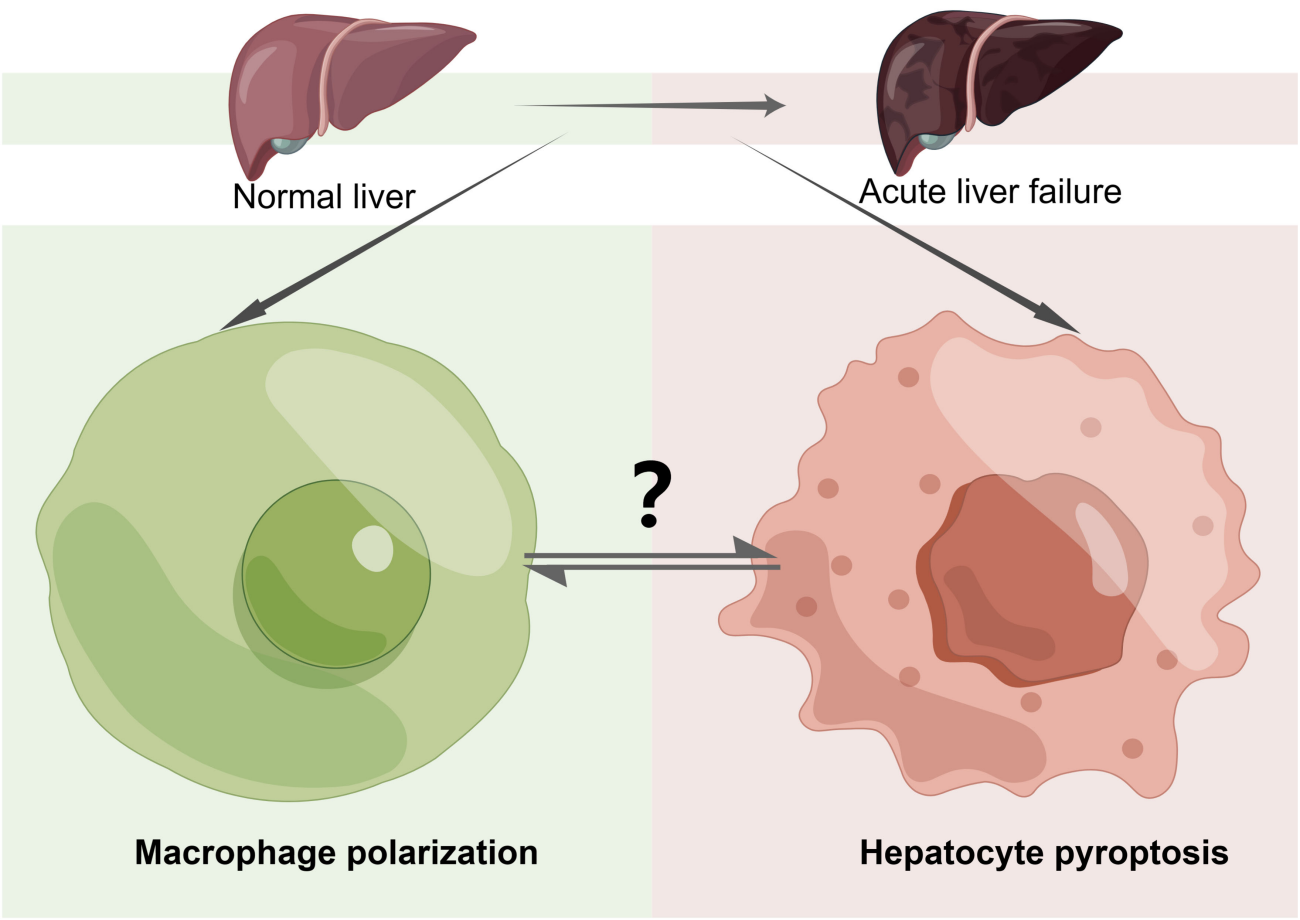
The Crosstalk of Pyroptosis and Macrophage Polarization in ALF. Macrophage polarization and hepatocyte pyroptosis play important roles in acute liver failure (ALF). The mutual crosstalk and regulation between these two processes can impact the progression of the disease. However, the specific mechanisms involved require further study and exploration. “?”: there are many unknown relationships between macrophage polarization and hepatocyte pyroptosis in ALF that need to be further explored and investigated in the future, including the impact of their mutual regulation and constraints on disease progression in ALF as well as the specific pathways of action.

M2 macrophages play a crucial role in protecting the liver in ALF. They exhibit hepatoprotective effects by releasing the anti-inflammatory cytokine IL-10 and pro-fibrosis (149). Additionally, M2 macrophages exert hepatoprotective effects by expressing the Galectin-3 (GAL3) gene, which inhibits the expression of pyroptosis signaling proteins in ALF mice (150). Surely, the one-way regulatory mechanism is incomplete. Some studies have suggested that hepatocyte pyroptosis mediated by GSDMD can recruit macrophages to release inflammatory mediators through the upregulation of the monocyte chemotactic protein 1/CC chemokine receptor-2 (MCRP1/CCR2) signaling pathway, leading to the spread of the inflammatory response. Furthermore, immunohistochemistry of the liver showed a significant decrease in the expression of the macrophage-specific protein F4/80 in the D-GalN/LPS ALF mouse model with GSDMD knockout, compared to the wild type(WT) D-GalN/LPS ALF model (127). This phenomenon suggests that inhibiting GSDMD-induced pyroptosis can significantly decrease macrophage infiltration in ALF liver tissue. In a wore, all of this evidence suggests a positive feedback loop between macrophage polarization and hepatocyte pyroptosis, which can induce an inflammatory cascade response in ALF. Given these observations, further research is needed to understand the cellular crosstalk between macrophage polarization and hepatocyte pyroptosis and its contribution to the progression of ALF. Additionally, the complex signaling pathways between these two processes should be explored and confirmed through additional experiments (**Figure 5**).

## 6 Conclusion and future directions

The study of the hepatocyte pyroptosis pathway in ALF is still in its preliminary stage. All factors that induce liver injury led to pyroptosis, and the late stage of hepatocyte pyroptosis, accompanied by the release of inflammatory factors such as IL-1β and IL-18, is central to the exacerbation of ALF (132, 151). The inflammatory factors released from hepatocyte pyroptosis enhance the activation of the NF-κB signaling pathway in hepatocytes and macrophages, thereby further promoting the inflammatory response. At the same time, the release of pro-inflammatory factors can recruit mononuclear macrophages in the body, leading to a more severe inflammatory response and ultimately causing massive hepatocyte death (22, 152). The suggestion of improving liver damage and inflammatory response by inhibiting pyroptosis in ALF has also been the focus of several studies. However, inhibiting pyroptosis is equivalent to compromising the system’s ability to clear microorganisms, which can potentially lead to opportunistic infections and even the development of adverse outcomes like bacteremia and sepsis. Therefore, further exploration is needed in the therapeutic strategy of treating ALF by inhibiting pyroptosis. This is important to consider due to the local intrahepatic damage and the systemic state. The aim is to find more precise methods of targeting intrahepatic pyroptosis.

The role of hepatic macrophages in ALF has been controversial. It is mentioned in our paper that macrophage activation is influenced by various factors in the immune microenvironment and can polarize in either a pro-inflammatory or an anti-inflammatory direction. However, polarized macrophages can still switch their polarization depending on the cytokines and mediators present in the immune microenvironment. Depending on the stage of ALF disease, macrophages can either perpetuate inflammation or promote its remission. These contradictions and inconsistencies are attributed to the diversity of macrophage subtypes. For this reason, regulating the direction of macrophage polarization has become a therapeutic approach in the study of ALF. However, therapies targeted at macrophages for the treatment of ALF carry certain risks and challenges. Firstly. However, promoting the anti-inflammatory effects of macrophages, they can improve liver damage and disease progression in ALF, but at the same time, they can also facilitate the spread of pathogens in the system and increase susceptibility to infection. Secondly, by promoting the pro-inflammatory effects of macrophages, it can effectively inhibit opportunistic infections and remove foreign pathogens. However, it can also easily induce a severe inflammatory response, resulting in the release of inflammatory mediators that are dispersed throughout the system via the bloodstream. This can lead to multi-organ damage and even failure. The contradictions of the approaches described for macrophage-targeted therapy highlight the challenges of immunotherapeutic strategies in ALF. The local hepatic immune microenvironment and the systemic immune microenvironment mutually regulate and influence each other. Anti-inflammatory therapy in the local hepatic immune microenvironment is effective in alleviating liver damage and repairing tissue. However, it has the opposite effect in controlling systemic pathogen dissemination. The timing of targeting application to induce different macrophage polarization directions needs to be precisely controlled, especially in order to clarify the function and composition of macrophages at different stages of ALF in humans.

Combining the signaling pathways and mechanisms of macrophage polarization and pyroptosis mentioned above, it is clear that several signaling pathways are shared between the two. For example, the TLR/NF-κB pathway not only plays an important role in M1 polarization but also induces cell pyroptosis (27, 67). In addition, several studies have also implicitly suggested that there is a reciprocal regulation between macrophage polarization and pyroptosis. For example, the knockdown of CD38 promotes the expression of molecules related to M1 polarization and also triggers more severe cell pyroptosis. CD38 serves as a common regulatory mediator of both processes (122). The interaction between macrophage polarization and pyroptosis still requires further investigation and exploration. In particular, the roles of both factors are crucial to the development of the disease. However, it is unclear whether they are mutually independent, whether they regulate each other, or if they even trigger a more severe inflammatory response through positive feedback. This assessment cannot be made without further experiments and studies to confirm.

In summary, this paper describes the mechanisms and regulatory pathways of macrophage polarization and pyroptosis in ALF. It also explores the interplay between macrophage polarization and pyroptosis. Fundamentally, macrophage polarization and pyroptosis in ALF are significant factors that have been extensively studied in recent years. However, there are still numerous unanswered questions that require further exploration. It is hoped that future research will address these questions, leading to new insights into the pathogenesis and therapeutic strategies for ALF.

## Author contributions

DX: Conceptualization, Writing – original draft. SO: Conceptualization, Writing – review & editing.
